# Healthy life skills and related factors among university students: a cross-sectional study in Istanbul, Turkey

**DOI:** 10.1186/s41043-023-00481-4

**Published:** 2023-12-06

**Authors:** Mahruk Rashidi, Funda Karaman, Gülay Yildirim, Aslı Genç, Sultan Çakmak, Ebru Durusoy, Buse Saygin Şahin, Nurten Elkin

**Affiliations:** 1 Faculty of Health Sciences, Istanbul Gelisim University, Istanbul, Turkey; 2 Keşan Hakkı Yörük School of Health, Trakya University, Edirne, Turkey; 3 Istanbul Esenyurt University, Istanbul, Turkey; 4 Graduate School of Health Sciences, Bursa Uludağ University, Bursa, Turkey; 5 Institute of Graduate Studies, Istanbul University- Cerrahpasa, Istanbul, Turkey

**Keywords:** Health behaviors, Life style, Nutrition, Physical activity, Prevention, Tobacco, Alcohol

## Abstract

**Background:**

Chronic non-communicable diseases (NCDs) are the leading cause of global deaths. University students with unhealthy lifestyle constitute a high-risk group for NCDs. Evaluating and developing healthy behaviors during this period is very important for future health outcomes. This study was conducted to determine healthy life skills in university students.

**Methods:**

A cross-sectional study was conducted at a university, and data were collected between January and March 2023 in Istanbul. Data were collected using the healthy living skills scale in University Students and the Personal Information Form. Normal distribution conditions of the data were checked with the Kolmogorov–Smirnov test. According to data distribution, data with normal distribution were analyzed using parametric statistics including t-test, ANOVA and Chi-square tests and data with non-normal distribution were analyzed using non-parametric tests including Mann–Whitney U-test and Fisher’s exact test. Logistic regression test was used to determine predictor variables.

**Results:**

The average score for healthy life skills was 63.5 out of 84. Significant differences were found in the scores based on marital status, economic income, social security insurance coverage, and educational grade (*p* = 0.03, *p* = 0.001, *p* = 0.004, *p* = 0.04, respectively). Students who reported alcohol and smoking consumption had lower scores (60.8 ± 12) out of 84.

**Conclusion:**

The study revealed that university students in Istanbul possess a satisfactory level of healthy life skills. By providing social support, such as expanding the coverage of social security insurance and establishing conducive educational environments, while also paying attention to the influence of peers on students, we can contribute to the development of healthy life skills in university students.

## Introduction

According to the World Health Organization (WHO), chronic non-communicable diseases (NCDs) account for 74% of deaths worldwide [1, 2]. Several factors have been associated with NCDs, unhealthy eating habits, tobacco and alcohol consumption, and physical inactivity being the most extensively studied risk factors [2]. Unhealthy diet and physical inactivity, often linked to lifestyle choices, are among the leading risk factors for non-communicable diseases [3]. Chronic diseases are commonly caused by long-term unhealthy habits and behaviors [4].

University students have been identified as a high-risk population for NCDs [5]. This phase of life represents a transitional period during which students develop habits that can influence their health throughout adulthood. As they gain more independence from their parents, they assume greater control over their daily choices regarding food, sleep, health management, physical activity, and engagement in harmful habits like smoking or excessive drinking [6]. University students face numerous challenges as they navigate the transition from adolescence to adulthood. During this critical phase, adopting and maintaining healthy behaviors becomes crucial for their overall well-being, academic success, and future quality of life.

The university years are considered a time when students may be exposed to health-related problems. They encounter new experiences, increased independence, changes in social circles, academic responsibilities, and greater accessibility to alcohol or drugs [7]. Studies have shown low percentages of health-promoting behaviors among university students [8].

Moreover, university students, who have recently entered adulthood, often overlook their health due to their relative youth and well-being. Consequently, they are more prone to engaging in behaviors that pose a risk to their health, such as inadequate physical activity, unhealthy eating habits, alcohol consumption, and smoking. Additionally, the behaviors of university students are highly influenced by their peers and environment [9].

The habits formed during this period can have lasting effects on their health in later years [10]. Furthermore, they are the leaders, decision-makers, and parents of tomorrow [11]. A healthy lifestyle developed during this time can lay the foundation for better health in middle age, reducing the prevalence of suboptimal health and delaying the onset of chronic diseases [9].

Empowering university students with healthy life skills enables them to make informed decisions, adopt healthy behaviors, and actively promote their own health and that of their communities. These skills encompass a range of abilities that enable individuals to adopt and maintain healthy behaviors. By developing and practicing healthy life skills, individuals can effectively manage stress, enhance self-care routines, and overcome challenges in their daily lives [12]. This empowerment contributes to improved health outcomes and overall well-being.

Furthermore, the university environment exposes students to diverse peer networks, making peer influence and social norms powerful determinants of health behaviors. Students often emulate the behaviors and habits exhibited by their peers, leading to either the adoption or rejection of healthy behaviors. Research has shown that positive peer influence and perceived social norms are positively associated with healthy dietary choices among university students [13].

Therefore, it is necessary to make efforts to improve health-promoting behaviors among university students. Promoting healthy behaviors is crucial for their overall well-being, academic success, and long- term health outcomes. Understanding their healthy life skills, behaviors, and related factors is essential in designing effective strategies to support and empower students in adopting and maintaining healthy lifestyles. By doing so, we can contribute to their well-being and create a healthier society.

University students with unhealthy lifestyle constitute a high-risk group for chronic non-communicable diseases. The aim of this study was determined healthy life skills in university students. Evaluating and developing healthy behaviors during this period is very important for future health outcomes. It is thought that the results of this study will provide essential data for programs to be planned to raise students' awareness about healthy lifestyle behaviors and increase their quality of life.

Questions expected to be answered in the research;

What are the identifying characteristics of university students?

What is the healthy living skills scale score of university students?

Is there a relationship between the descriptive characteristics of university students and their healthy living skills scale scores?

## Materials and methods

The study was a descriptive-analytic cross-sectional study conducted at Istanbul Gelisim University in Istanbul, Turkey. The study received approval from the Ethics Committee for Research with a specific code: 2023-04-95. Data were collected between January and March 2023 in Istanbul. Data were collected using the healthy living skills scale in University Students and the personal information form. Normal distribution conditions of the data were checked with the Kolmogorov–Smirnov test. According to data distribution, data with normal distribution were analyzed using parametric statistics including t-test, ANOVA and Chi-square tests and data with non-normal distribution were analyzed using non-parametric tests including Mann–Whitney U-test and Fisher’s exact test. Logistic regression test was used to determine predictor variables. The sample number of the study was calculated using the sampling formula with a known population. The sample of the universe consisting of 17,000 students was determined as 546. Students were informed that the data would be kept confidential and that they could leave the study whenever they wanted and without facing any negative consequences. Informed consent was obtained. Being a university student, knowing Turkish and being a volunteer are the inclusion criteria for the study. Participants were from Faculty of Engineering, Law and Health Sciences at the university.

The researchers aimed to assess healthy life skills among university students using a questionnaire developed by Genc in Turkey [14]. The questionnaire consisted of 21 items, each presented as a four-point Likert scale statement, ranging from 1 for “Strongly disagree” to 4 for “Strongly agree.” The minimum score could be 21, and the maximum 84.

The questionnaire assessed four subscales related to healthy life skills: importance given to health, healthy nutrition, access to health-related resources, and health priority. Each subscale had a different number of questions and a unique scoring range.

*The importance given to health* was assessed through 8 questions on a 4-point Likert scale. For each question, Scores ranged from 1 for strongly disagree to 4 for strongly agree. In this section, the minimum score could be 8, and the maximum 32.

*The healthy nutrition* was assessed through 5 questions on a 4-point Likert scale. For each question, Scores ranged from 1 for strongly disagree to 4 for strongly agree. In this section, the minimum score could be 5, and the maximum 20.

*The access to health-related resources* was assessed through 5 questions on a 4-point Likert scale. For each question, Scores ranged from 1 for strongly disagree to 4 for strongly agree. In this section, the minimum score could be 5, and the maximum 20.

*The health priority* was assessed through 3 questions on a 4-point Likert scale. For each question, Scores ranged from 1 for strongly disagree to 4 for strongly agree. In this section, the minimum score could be 3, and the maximum 12.

The reliability of the questionnaire was assessed using Cronbach's alpha, resulting in a calculated value of 0.76, indicating acceptable reliability. The validity of the questionnaires employed in this study was obtained through experts’ comments on the questionnaires’ items.

Before administering the questionnaires, the researchers provided an explanation of the study objectives to the participants and obtained explicit verbal consent. All personal information collected from the subjects was treated as confidential.

Data analysis was performed using SPSS ver. 20 (SPSS Inc., Chicago, IL, USA). Categorical variables were presented as frequencies, while healthy life skill scores were treated as continuous variables and described using mean and standard deviation (SD). Various statistical tests were employed, including Chi-squared tests, two-tailed between-subject t-tests, Mann–Whitney U-test, one-way ANOVA test, post hoc tests, multiple regression, and univariate analysis, to determine differences between samples and explore relationships between variables. The significance level for all statistical tests was set at *p* < 0.05.

## Results

A total of 547 students participated in the study. The mean age of the participants was 21.6 ± 2.9 years, with a standard deviation of 2.9. Out of the participants, 281 (51.4%) were males, while 266 (48.6%) were females. Regarding marital status, the majority of students, 529 (96.7%), were single, while 18 students (3.3%) were married. In terms of academic standing, 286 students (52.3%) were lowerclassmen, and 264 students (47.7%) were in their senior year. Additionally, the majority of students, 387 (70.7%), hailed from urban areas. A total of 401 students (73.3%) considered their economic status as sufficient, while 403 students (73.3%) had health insurance coverage. Additional file 1: Table S1 presents the basic information of the participants.

The average score of the participants' healthy life skills was 63.5 ± 11.7. The results revealed a significant difference in scores based on the participants' marital status, economic income, social security insurance coverage, and educational grade. The participants with quiet well had the highest average score in healthy life skills (67.6 ± 11.7), whereas those with insufficient income had the lowest score (58.4 ± 13.8) (*p* < 0.001).

Married participants exhibited a higher average life skills score compared to single participants (68.8 ± 7.2 vs. 63.3 ± 11.7) (*p* = 0.03). Furthermore, senior year students obtained higher average scores in the field of healthy life skills compared to junior year students (66.2 ± 11.1 and 63.1 ± 11, respectively) (*p* = 0.04).

Also, participants with social security insurance coverage obtained higher scores in healthy life skills compared to students without social security insurance, respectively (64.4 ± 11.2, 61 ± 12.6) (*p* = 0.004). No significant differences were found in healthy life skills scores between participants with underlying diseases and those without, as well as between participants who required constant medication and those who did not (*p* > 0.05) (Table 1).

**Table 1:** Comparation healthy life skill scores by select socio-demographic variables

	Mean ± SD	*p* value
Total mean of healthy life skill sore	63.5 ± 11.7	**p** value
Sex		
Male	63.3 ± 11.8	0.63
Female	63.8 ± 11.5	
Mother level of education		
Illiterate	64.2 ± 10.3	0.6
Primary school	63.3 ± 11.7	
High school	64.2 ± 12	
University	61.7 ± 12.3	
Father level of education		
Illiterate	63.5 ± 9	0.83
Primary school	63.6 ± 11	
High school	63.1 ± 11	
University	64.3 ± 11.9	
Marital status		
Single	63.3 ± 11.7	**0.03**
Married	68.8 ± 7.2	
Grade		
Lowerclassman	63.1 ± 11	**0.04**
Senior year student	66.2 ± 11.1	
Place of hometown		
Rural	62.1 ± 11.9	0.15
Urban	64.1 ± 12.5	
Economic status		
Quiet well	67.6 ± 11.7	** < 0.001**
Insufficient	58.4 ± 13.8	
Sufficient	63.7 ± 10.9	
Insurance		
Yes	64.4 ± 11.2	**0.004**
No	61 ± 12.6	
Live with parent		
Yes	63.7 ± 11.3	0.55
No	62.9 ± 12.4	
Illness history		
Yes	63 ± 12.5	0.64
No	63.6 ± 11.5	
Need to use drug for illness		
Yes	62.9 ± 13.6	0.64
No	63.6 ± 11.3	
Only child		
Yes	63.7 ± 11.3	0.55
No	62.9 ± 12.4	

The significance level was accepted as p < 0.05 for all analyses. Since there is a significant difference, it is written in bold

Furthermore, 132 (24.1%) participants reported being smokers, while 114 (20.8%) reported alcohol use. Simultaneous use of alcohol and cigarettes was reported by 72 (13.5%) participants. Participants who used both alcohol and smoking had a lower score in healthy life skills (60.8 ± 12) compared to those who did not (64.3 ± 11) (*p* < 0.05) (Additional file 1: Table S2).

The mean scores of students in different subscales of life skills were estimated. The mean score for the importance given to the health subscale was 25.8 ± 4.6 out of 32. The mean score for healthy nutrition was 14.3 ± 3.4 out of 20. The mean score for access to health-related resources was 12.6 ± 3.8 out of 20. The mean score for health priority was 10.7 ± 1.7 out of 12.

The mean scores of students in different subscales of life skills were compared according to demographic and behavioral variables. The results showed that the scores of students in the health priority subcategory were statistically higher among senior year students (11.1 ± 1.4), students with a fairly good economic status (11 ± 1.4), and students with social insurance (10.8 ± 1.5) (*p* < 0.05).

The scores of students in the subscale of importance given to health were statistically higher among students with a quite good economic status (27.1 ± 4.3) and those with social insurance (26.01 ± 4.3), and lower among students who smoke (24.5 ± 4.9) and consume alcohol (24.6 ± 4.9) (*p* < 0.05).

The scores of students in the subscale of healthy nutrition were statistically higher among students with an economic status and social insurance (*p* < 0.05). The scores of students in the subscale of access to health-related resources were statistically higher among senior year students and students with a quite good economic status, and lower among students who smoke and consume alcohol (*p* < 0.05) (Table 2).

**Table 2:** Comparison of students healthy life skill score in four dimension of healthy life scale according to demographic and behavioral characteristics

	Mean(SD)			
	The importance given to health	Healthy nutrition	Access to health-related resources	Health priority
Total score	25.8 (4.6)	14.3 (3.4)	12.6 (3.8)	10.7 (1.7)
Highest and lowest obtainable score	8–32	5–20	5–20	3–12
Age				
Coefficient of correlation	0.07	0.11	0.11	0.08
*p* value	0.06	0.008	0.006	**0.03**
Grade				
Lowerclassman	25.6 (4.7)	14.2 (3.4)	12.5 (3.8)	10.6 (1.7)
Senior year student	26.6 (4.3)	14.9 (3.5)	13.4 (3.5)	11.1 (1.4)
*p* value	0.12	0.12	0.05	**0.03**
Economic status				
Quiet well	27.1 (4.3)	15.9 (3.2)	13.4 (4.3)	11 (1.4)
Insufficient	23.8 (5.6)	12.5 (3.7)	12 (4.1)	10 (2.1)
Sufficient	25.9 (4.4)	14.4 (3.4)	12.5 (3.6)	10.8 (1.6)
*p* value	0.001	0.001	0.06	**0.001**
Social insurance				
Yes	26.1 (4.3)	14.6 (3.4)	12.7 (3.8)	10.8 (1.5)
No	24.7 (5.4)	13.6 (3.5)	12.1 (3.8)	10.4 (2)
*p* value	**0.002**	0.002	0.1	**0.01**
Smoking				
Yes	24.5 (4.9)	13.9 (3.7)	12.06 (4.2)	10.2 (2)
No	26.2 (4.5)	14.5 (3.4)	12.8 (3.7)	10.9 (1.5)
*p* value	**0.001**	0.14	**0.05**	**0.001**
Alcohol consumption				
Yes	24.6 (4.9)	14 (3.5)	11.9 (3.9)	10.2 (1.9)
No	26.1 (4.5)	14.4 (3.4)	12.8 (3.8)	10.8 (1.6)
*p* value	**0.002**	0.2	**0.03**	**0.001**

The significance level was accepted as p<0.05 for all analyses. Since there is a significant difference, it is written in bold

Regression analysis between healthy life skill scores as the dependent variable and demographic and behavioral factors as independent variables showed that having social security insurance and not smoking are predictors of healthy life skills (*p* = 0.004, *p* = 0.04) (Table 3).

**Table 3:** Factors associated with healthy life skills among participants (multiple regression)

Variable	B	Beta	Sig
Education grade	2.59	0.74	0.081
Economic status	1.23	0.74	0.08
Insurance social	− 0.122	− 3.25	**0.004**
Smoking	2.64	0.097	**0.04**
Alcohol consumption	2.49	0.087	0.074
Age	0.423	0.108	0.059
Mariage	− 0.362	− 0.006	0.919

The significance level was accepted as p<0.05 for all analyses. Since there is a significant difference, it is written in bold

## Discussion

Many studies have been conducted to investigate health literacy among university students, but few studies have investigated healthy life skills. The World Health Organization (WHO) defines life skills as "abilities for adaptive and positive behavior that enable an individual to deal effectively with the demands and challenges of everyday life" [15]. These sets of skills help individuals develop psychosocial competence and empower people to have control over what they do [15]. This study examined university students' healthy life skills and related factors in Turkey.

The results of the study revealed that the mean score for students' healthy life skills was 63.5 ± 11.7 out of a maximum score of 84. This indicates that, on average, the participants demonstrated a favorable level of healthy life skills. The reasons for this could be attributed to the curious nature of youth and their eagerness and interest in learning new things [16]. Developing healthy life skills helps students make more reasoned and intentional choices, enabling them to practice healthy behaviors, resulting in fewer diseases. The results showed a positive correlation between the age of students and their healthy life scores. This correlation also was seen between the four subscales of healthy life skills and students' age. It seems that as age increases, their health literacy and life skills develop.

Being a woman positively influences the health literacy level [17]. In this study, women had slightly higher scores than men, although not significantly. The lack of gender significance difference in the healthy life skill score can be due to the high access to digital information sources in recent years and the increase in the learning and ability of both sexes in healthy life skills. The capacity of digital tools to facilitate connectivity and information exchange is particularly important [18]. Evidence worldwide indicates the proven effectiveness of life skills training in bringing positive change among vulnerable populations [16].

The self-reported consumption of alcohol and smoking was 20% and 24% among participants, respectively. The study also showed that there was a difference in the score of healthy life skills between individuals exposed to risky behaviors, including smoking (and alcohol consumption, and those without such unhealthy behaviors (60.8 ± 12.6, *p* = 0.004, 60.7 ± 12.1, *p* = 0.005) (Additional file 1: Table S2). The mean score of students' healthy life skills was lower in smoking and alcohol consumer students than in students without these unhealthy behaviors. Low levels of life skills are known to develop high-risk behaviors that lead to long-lasting health and social consequences [15].

Life skills are psychosocial and interpersonal "abilities for adaptive and positive behavior that enable individuals to deal effectively with the demands and challenges of everyday life" [12]. This implies that promoting healthy life skills is effective in reducing unsafe behaviors such as smoking and alcohol consumption, and consequently reducing non-communicable diseases in later years. A study conducted in the USA found that respondents who had lower-risk lifestyles, such as non-smoking, having a healthy weight, adequate physical activity, moderate alcohol consumption, and a balanced diet, lived longer [19].

The results of the study showed that students in higher grades have higher scores in healthy life skills. This result is similar to studies done by Rabab and Ozen, who reported that students in higher grades have better health literacy levels compared to those in lower grades [17, 20]. Higher healthy life skill scores were observed in students with a quite well economic status. This result was shown in all four dimensions of the scale (67.6 ± 11.7, *p* < 0.001). It is because individuals with higher socioeconomic status tend to have better access to health-promoting resources, such as nutritious food, recreational facilities, and healthcare services.

The highest Healthy Life Skill scores for university students in this study were observed among the married students. It is probable that married students are in a supportive environment that enhances their ability to learn healthy life skills. Other sociodemographic factors such as parents' education level, living arrangements with parents, and place of residence did not yield significant results. Although these factors might have had some impact, only a weak relationship could be established. Specialization can be an influencing factor [21], but in this study, no difference was shown between the healthy life scores of health-related students and students from other sectors.

A study conducted by Dan Ping found that health-related students had better scores [9]. In Asia, the level of health literacy among 224 students from two faculties (Science and Health Sciences) was measured. The study findings indicated that 93% of the students had insufficient health literacy [22]. Another study carried out in Turkey among Nursing students concluded that 29.3% of the students had an insufficient level of health literacy [23]. The study conducted by Budhathokia et al. found that students had moderate skills to manage their health [24].

Health-related students are future healthcare providers, and they will play a key role in both modeling lifestyle and teaching healthy choices to clients [7, 25]. Therefore, these students should have a higher level of healthy life skills than other students. The health-related students, who have been exposed to more health-related courses and would become health providers in the future, were expected to have higher scores. Unfortunately, this study didn't show any difference between the two groups. This issue may arise from students being influenced by other factors, such as their friends and peers.

Having social support has always had a positive effect on improving people’s health [26, 27]. The results of this study also indicate the positive effect of having health insurance on students' healthy life skills (*p* = 0.004). As the study results showed, having social security insurance was one of the predictive factors for the level of healthy living skills in students.

The strengths of the research are that it is conducted by a multidisciplinary team, has a sufficient number of samples, and collects study data through valid and reliable tools. The limitations of the study are that the study results can only be generalized to the determined sample group and that the data are based on self-report.

## Conclusion

The study revealed that university students in Istanbul possess a satisfactory level of healthy life skills. By providing social support, such as expanding the coverage of social security insurance and establishing conducive educational environments, while also paying attention to the influence of peers on students, this study can contribute to the development of healthy life skills in university students. Empowering students with healthy life skills is crucial for enhancing their overall well-being and long-term health outcomes.

## Data Availability

The datasets used and/or analyzed during the current study are available from the corresponding author on reasonable request.
