# Correction to: Healthy life skills and related factors among university students: a cross‑sectional study in Istanbul, Turkey

**DOI:** 10.1186/s41043-024-00574-8

**Published:** 2024-06-17

**Authors:** Mahruk Rashidi, Funda Karaman, Gülay Yildirim, Asli Genç, Sultan Çakmak, Ebru Durusoy, Buse Saygin Şahin, Nurten Elkin

**Affiliations:** 1 Faculty of Health Sciences, Istanbul Gelisim University, Istanbul, Turkey; 2 Keşan Hakkı Yörük School of Health, Trakya University, Edirne, Turkey; 3 Istanbul Esenyurt University, Istanbul, Turkey; 4 Graduate School of Health Sciences, Bursa Uludağ University, Bursa, Turkey; 5 Institute of Graduate Studies, Istanbul University-Cerrahpasa, Istanbul, Turkey

**Correction to: J Health Popul Nutr 42, 137 (2023).**

10.1186/s41043-023-00481-4

Following publication of the original article [1], the authors identified an error in the affiliation of Aslı Genç.

The incorrect affiliation for author Aslı Genç reads: Faculty of Health Sciences, Istanbul Gelisim University, Istanbul, Turkey

The correct affiliation for author Aslı Genç is: Istanbul Esenyurt University, Istanbul, Turkey

The authors affiliations have been updated above and the original article [1] has been corrected.
